# ST-Elevation Myocardial Infarction: A Simulation Case for Evaluation of Interprofessional Performance in a Hospital

**DOI:** 10.1155/2019/7562637

**Published:** 2019-10-07

**Authors:** Hadiki Habib, Eka Ginanjar, Arif Mansjoer, Septo Sulistio, Imamul A. Albar, Radi M. Mulyana

**Affiliations:** ^1^Emergency Unit, Cipto Mangunkusumo Hospital, Central Jakarta 10430, Indonesia; ^2^Cardiovascular Comprehensive Service, Cipto Mangunkusumo Hospital, Central Jakarta 10430, Indonesia

## Abstract

**Introduction:**

Interprofessional collaboration between units in a hospital is essential in order to reach desired time for primary percutaneous intervention (PCI) in acute ST-Segment Elevation Myocardial Infarction (STEMI) cases. We developed a simulation to engage various medical and nonmedical staff in interprofessional and interunit team collaboration.

**Method:**

We used a scenario in this simulation. Beginning in the emergency department, it detailed a 50-year-old male presenting with progressive chest pain since 7 hours before admission. The emergency team directly examined the patient, and STEMI diagnosis was made, followed by sending the patient to the cardiac catheterization laboratory to undergo primary PCI. A resuscitation kit was required for the simulation. An evaluation sheet was prepared to evaluate every step of patient management. Three judges observed the simulation. At the end of the simulation, debriefing was done, and recommendation for the simulation was discussed. Besides medical activities during patient management, interprofessional communication, administration activities, consultations, and handover process were also evaluated.

**Results:**

The team achieved the appropriate door-to-electrocardiogram (ECG) time in 8 minutes, but overall target was delayed since door-to-skin puncture time was reached in 110 minutes. Some factors that contributed to these conditions were long waiting time during patient admission, several attempts for telephone consultation to the cardiologist, and prolonged admission process in the cardiac catheterization laboratory.

**Conclusions:**

The simulation was well received by both participant and our institution, stating that it is a valuable resource for developing interdisciplinary learning program. This simulation also contributed to the development of the clinical pathway, STEMI protocol, in our institution.

## 1. Introduction

ST-Segment Elevation Myocardial Infarction (STEMI) is a time-sensitive cardiovascular emergency, in which early definitive treatment from fibrinolytic or primary percutaneous intervention (PCI) will improve the clinical outcome [1–3].

The National Health Survey of Indonesia [4] in 2006 showed that cerebro-cardiovascular diseases are the leading cause of death in Indonesia, supported by a 13-year cohort study [5]. Harapan Kita, Indonesia National Cardiovascular Hospital [6] reported there were 2103 cases of acute coronary syndrome in 2008, and 31.1% of the cases were STEMI. Primary PCI was done in 29% of STEMI cases, fibrinolytic in 12% cases, and nonreperfusion strategy in 59% cases.

As the previous study identified [6], there were five potential problems in managing STEMI in daily practices in Jakarta such as patient delay, diagnosis and treatment decision delay, transportation delay, lack of collaboration between hospitals and doctors, and also lack of ambulance organization.

Based on those identified problems and targets that the guideline had already proposed, the emergency unit and the comprehensive cardiac service unit of Cipto Mangunkusumo Hospital performed an *in situ* STEMI case simulation in a multidisciplinary emergency unit. The target participants were medical professionals such as emergency physician, internist, cardiologist, emergency nurse, cardiac laboratory nurse, pharmacist, radiographer, and those of nonmedical staff such as security officer, front desk officer, cashier, administration officer, and transporter.

Lectures and focus group discussions, aimed to introduce the guideline of current STEMI management practices, were delivered prior to the simulation for the participants. Real life situation showed various interprofessional interactions while managing a STEMI case. This proves the need for excellent teamwork for optimal patient outcome.

There were three main objectives of the simulation: to provide teamwork learning, to identify pearls and pitfall of clinical management of STEMI from emergency unit arrival until disposition to an intensive cardiac care unit, and to educate the roles of medical and nonmedical staff involved in STEMI management. This simulation method was developed based on the framework provided by the Interprofessional Education Collaborative (IPEC) that includes values and ethics, roles and responsibilities, communication, and teamwork.

There is a published case of STEMI in MededPORTAL [7] that can complement this case. Our simulation is unique in its particular emphasis on collaborative teamwork, communication skills between medical and nonmedical staff, and administrative process, so that this team can achieve the appropriate time to definitive treatment with door-to-balloon time ≤90 minutes.

This resource can be used by other general hospitals for training the multidisciplinary staff about the appropriate management of STEMI from triage counter to the cardiac catheterization laboratory. Feedback obtained from this simulation can be used as a source for quality improvement of the in-hospital emergency system regarding STEMI management in other hospitals.

## 2. Materials and Methods

### 2.1. Development

The simulation was targeted to medical and nonmedical staff in the emergency unit and cardiac center. The simulation was done during office hour. Prerequisite knowledge about signs and symptoms of STEMI was needed for the actors in this simulation; the emergency medical officer who participated in the simulation already knows the scenario but still had to act as if it was in real condition. Scenario was made and evaluated before the simulation was performed. See [Supplementary-material supplementary-material-1] in Supplementary Materials for the complete scenario template. Two experts evaluated the scenario: one emergency physician from the emergency unit and one cardiologist from comprehensive cardiovascular service. Table-top exercise was done twice in 20th and 22nd of December 2016 to make sure that every actor/actress understood the scenario and had proper action to achieve high engineering and psychological fidelity simulation as stated by Adams et al. [8].

### 2.2. Equipment/Environment

All standard items of resuscitation kit, defibrillator, cardiac monitor, and cardiac medications were prepared. Specific ECG with STEMI case was prepared. See [Supplementary-material supplementary-material-1] in Supplementary Materials for the ECG. No specific moulage was required for the actor. The simulation was done *in situ*, begun in emergency unit triage, and ended right after the patient arrived in the cardiac catheterization laboratory.

### 2.3. Personnel

The team consists of an emergency medical officer, emergency physician, internal medicine resident, cardiologist, head nurse, associate nurses, triage officer, administration officers in the emergency unit and cardiac center, security, transporter, and cardiac catheterization laboratory nurses, with a total of 28 personnel. Cardiologist was there to simulate the consultation process. An emergency physician as a coordinator of the scenario who was familiar with the case was available within the simulation area to help any unexpected issue or troubleshoot, as well as providing information about ECG and laboratory result. All team members were able to discuss with the scenario coordinator to make decisions. The coordinator must not intervene the clinical management given to the patient during simulation. Two actors were prepared as a patient and the patient's family.

### 2.4. Implementation

Based on the scenario, the main information that the team members ought to know was the diagnosis of the case and the decision to perform primary PCI. Information about patient clinical symptoms and signs were only made available to the actors performing patient and his family. Nonmedical staff were informed about the simulation schedule and asked to perform the usual business.

On the simulation day, December 27, 2016, all the staff were on their position, the emergency unit and cardiac center. The patient and his wife entered the triage counter in the emergency unit, and the usual process was done from triage assessment and disposition to the red zone. In the red zone, the emergency medical officer, together with an internal medicine resident and emergency nurse, performed STEMI management. ECG was recorded, and its result was changed with STEMI ECG case that had been prepared prior to the simulation by the scenario coordinator. The internal medicine resident then contacted the cardiologist consultant to report on the patient's condition and asked for primary PCI approval. The patient's wife went to the administration counter for registration and insurance activation.

The patient was positioned on a bed in the semifowler position; nasal cannula oxygen was given 3 liters per minute, and IV catheter was inserted to the patient's vein placed at hand. Oral medication was prepared and placed on the table beside the bed. All the blank in medical records had to be filled completely, including inform consent for primary PCI.

After the decision for primary PCI was declared by the cardiologist consultant, the emergency unit head nurse then contacted the cardiac catheterization laboratory nurse to put the patient on the PCI schedule and contacted the cardiac center administration officer to send the patient's data. While waiting for instruction from the cardiac laboratory to send the patient, the nurse, emergency medical officer, and internal medicine resident prepared the patient for transfer, which consists of completing the transfer form, putting on a transport monitor, and preparing the oxygen transport device and emergency bag. The transportation process was done by an emergency medical officer, one emergency nurse, and one transporter to the cardiac catheterization laboratory.

The handover process was done in front of the cardiac laboratory entrance door between the emergency medical officer, emergency nurse, and cardiac nurse. The patient then moved from the previous bed to the cardiac laboratory bed. The simulation ended after the patient arrived at the cardiac catheterization room and was recorded in a video for debriefing purpose. See video [Supplementary-material supplementary-material-1] for the recorded simulation.

### 2.5. Assessment

An evaluation sheet was prepared to evaluate every step of the patient management. See [Supplementary-material supplementary-material-1] in Supplementary Materials for complete evaluation sheet. A team of judges consisting of a cardiologist, an emergency head nurse, and a cardiac laboratory nurse observed the simulation. Video recording was also replayed for retrospective evaluation and educational purposes. The judges reviewed the simulation and gave proper feedback and recommendation. There are two general standards for proper STEMI management: door-to-ECG and door-to-patient ready at the catheterization laboratory bed. Door-to-ECG is defined as time between patient presenting at triage counter and performing ECG. The standard for this is less than 10 minutes. Door-to-patient ready at the catheterization laboratory bed is defined as time between patient presenting at triage counter and patient being ready at catheterization laboratory. The standard for this is less than 90 minutes. A self-evaluation questionnaire was given to the participants after the simulation ended for feedback.

### 2.6. Debriefing

Debriefing session was conducted 3 days after the simulation. All the judges and emergency physician in charge lead the session for one hour. Further medical and administrative management were discussed and included the questions below:

- What is needed in STEMI management to diagnose right on time?
- Are there any unimportant or repetitive activities that could potentially delay STEMI management in the emergency unit?
- What should be done for the activation of the cardiac catheterization laboratory for primary PCI?
- How to have effective communication between the emergency unit and the cardiac catheterization laboratory to minimize the delay of primary PCI?
- How to perform effective transportation of a critically ill patient from the emergency unit to the cardiac catheterization laboratory?

## 3. Results

The simulation was performed one time *in situ*, with 28 personnel participating in it. Our team can approach appropriate door-to-ECG time in 8 minutes, but the overall target was not achieved where the door-to-patient ready at the catheterization laboratory bed was 110 minutes. Some factors that contribute to these conditions were the long wait in patient admission, prolonged administrative process regarding the activation of the cardiac catheterization laboratory, and time consuming phone consultation process to the cardiologist.

Case evaluation was completed by six people from the emergency medical officer, emergency nurses, internal medicine resident, and cardiac laboratory nurses. Response rate was 100 percent, 83.3% of which agreed that medical and administration scenario in simulation would be in accordance with daily condition. All responders agreed that the simulation could increase the knowledge of the administration process in the emergency unit and cardiac center.

Feedbacks were positive regarding the realistic nature of the case, especially when performing communication and administration processes. By doing this simulation, each participant learns to understand each unit challenge during STEMI case management. All responders agreed that mutual understanding of each person's role and responsibility in managing STEMI is important for teamwork. Findings and recommendations from the judges during debriefing process are available in [Supplementary-material supplementary-material-1] in Supplementary Materials.

Some selected comments from the participants were as follows:

  “I realized a lot of activities need to be completed by emergency unit personnel during initial management of STEMI case.”
  “I understand the administrative process that needs to be carried out from patient admission until definitive treatment, primary PCI, is done.”

## 4. Discussion

Simulation is extensively practiced in medicine as one of the educational methods. We also used the simulation in this report as an evaluation tool. We can identify the knowledge and medical skills every person has and how they contribute to the team. In addition, we also can qualitatively measure how a complex interunit activity in a hospital tries to comply with the guideline in managing STEMI. This method also can give a new perspective for every unit that is responsible for STEMI management about barrier and defect in the healthcare systems.

The simulation case was well received by both participants and our institution. It is a valuable resource for interdisciplinary learning program especially those of emergency medicine, internal medicine, and cardiology background, as well as other medical and nonmedical staff in the hospital. The *in situ* simulation was done at very low cost. Most money was spent for administration and documentation purposes.

Lessons learned by the participants by doing this simulation were to acknowledge some activities that have contribution to delay the STEMI management, such as long waiting time during patient admission, several attempts for telephone consultation to the cardiologist, and prolonged admission process in the cardiac catheterization laboratory.

The outcome of the simulation is the development of an integrated emergency clinical pathway, STEMI protocol, in our institution. The evidence supports that appropriate reperfusion treatment by fibrinolysis or primary PCI in STEMI is a significant predictor of lower mortality as Berger et al. mentioned [9]. Jacobs et al. stated that an interdisciplinary written protocol should be developed to achieve expected time response for STEMI reperfusion management [2].

Clinical audit was also conducted, the so called 5D time consisting of *Door, Diagnosis, Decision, Delivery,* and *Definitive.* Door is defined as admission time to triage counter. Diagnosis is defined as completion time of ECG testing. Decision is defined as time when decision of primary PCI or fibrinolytic was made. Delivery is defined as time of patient transfer to the cardiac catheterization laboratory. Definitive is defined as time of balloon inflation. However, later in 2017, this definition was changed to time of cross wire according to the European Society of Cardiology Guideline [3, 10].

Since this simulation was ran *in situ* and in a general hospital, the result and recommendation can apply to other general hospitals that have cardiac catheterization laboratory. The simulation method can be used to train staff from these two units, the emergency unit and the cardiac laboratory unit, so that they can reach an equal understanding of each other's work.

Some strengths of our simulation are the availability of validated scenario, *in situ* practice, being reproducible, and has a complexity element. *In situ* simulation practice contributes to increasing the fidelity and the team dynamics, according to the recommendation published in MedEdPORTAL [11]. We also evaluated the simulation using a checklist evaluation sheet and doing proper debriefing process. Some weaknesses of our simulation are the lack of surprise element because the scenario was already learned during table-top exercise by some participants, and the simulation was only performed once.

## 5. Conclusions

The simulation was well received by both participant and our institution, stating that it is a valuable resource for developing interdisciplinary learning program. This simulation also contributed to the development the clinical pathway, STEMI protocol, in our institution.

## Data Availability

The data used to support the findings of this study are included within the supplementary information files.

## Supplementary Materials

Supplementary Materials S1: simulation case template; S2: visual stimulation (STEMI ECG); S3: evaluation sheet; S4: simulation video; S5: debriefing material.
