# Supplementary material for: ST-Elevation Myocardial Infarction: A Simulation Case for Evaluation of Interprofessional Performance in a Hospital
Source: Emerg Med Int. 2019 Oct 7;2019:7562637. doi: 10.1155/2019/7562637 (PMC6800974; doi:10.1155/2019/7562637)
Supplement: Supplementary Materials — S1: simulation case template; S2: visual stimulation (STEMI ECG); S3: evaluation sheet; S4: simulation video; S5: debriefing material. [file 7562637.f1.zip › 7562637.f1/S1 Simulation case template.docx]

| **Appendix A: MedEdPORTAL Simulation Case Template**  **SIMULATION CASE TITLE: ST-Elevation Myocardial Infarct**  **AUTHORS: Hadiki Habib, MD** | |
| --- | --- |
| **PATIENT NAME: Mr. Musni**  **PATIENT AGE: 50 years old**  **CHIEF COMPLAINT: progressive chest pain** | |
|  | |
| **Brief narrative description of case**  *Include the presenting patient chief complaint and overall learner goals for this case* | a 50 years old man, came to the Emergency Department of General hospital at 10.00 am because of progressive chest pain start at 03.00 am. His wife accompanied him.  His main complaints were chest pain and breathlessness and diaphoresis. Mr. Musni had no history of chest pain before; he had uncontrolled hypertension and active smoker. No history of antiplatelet consumption or history of cardiac disease in his family. No history of allergies  Participants need to recognize abnormal vital signs, prioritize stabilization actions, initiate ECG testing, consult cardiologist, and start management of acute coronary syndrome. |
| **Primary Learning Objectives**  *What should the learners gain in terms of knowledge and skill from this case? Use action verbs and utilize Bloom’s Taxonomy as a conceptual guide* | By the end of this simulation case participants will be able to:   1. Understand the value of time sensitive in STEMI management 2. Understand each healthcare profession own role during collaborative approach to reach door to definitive treatment time in STEMI management 3. Demonstrate the role of non-medical staffs to reach door to definitive treatment time in STEMI management 4. Demonstrate communication skill with patient, families, and other health professionals that promote respect and empathy 5. Identify pearls and pitfall of clinical management of STEMI from emergency department arrival until disposition to a cardiac catheterization unit |
| **Critical Actions**  *List which steps the participants should take to successfully manage the simulated patient. These should be listed as concrete actions that are distinct from the overall learning objectives of the case.* | Specific Critical Actions   1. Triage process 2. Obtain ECG testing 3. Perform a brief focused history and exam 4. Giving double antiplatelet and sublingual nitrate for acute coronary syndrome 5. Consultation to cardiologist 6. Verbalize diagnosis with team members 7. Activate cardiac laboratory for primary PCI 8. Inform consent to patient and his wife 9. Perform administration and billing process in ED and cardiac center 10. Transport patient to cardiac laboratory 11. Handover process between Emergency medical officer and cardiac laboratory nurse |
| **Learner Preparation**  *What information should the learners be given prior to initiation of the case?* | Learners already know that the patient will come and diagnosis is STEMI |

| Initial Presentation | | | |
| --- | --- | --- | --- |
| **Initial vital signs** | BP 120/90 HR 100 times per minutes, RR 20x/minutes Temp 98, Oxygen Saturation 99% on O2 cannula 3 liter/minutes | | |
| **Overall Appearance**  *What do learners see when they first enter the room?* | Patient is middle aged male, anxiety appearing | | |
| **Actors and roles in the room at case start**  *Who is present at the beginning and what is their role? Who may play them?* | Confederate: Family Member (wife)  This member can provide additional history including history of uncontrolled hypertension and active smoker.  Confederate: cardiologist consultant  This role played by real cardiologist calling by the internal medicine resident. He will recommend primary PCI | | |
| **HPI**  *Please specify what info here and below must be asked vs what is volunteered by patient or other participants* | Chief Complaint: “pain and squeezed on the chest”  Information from patient (look anxious): “I’ve . . . been . . . . feeling . . .chest pain and breathlessness for a . . . little while. I really don’t feel good.”  Additional information (provide only when asked for):  Chest pain start suddenly at 03.00 am  Positive for diaphoresis | | |
| **Past Medical/Surgical History** | **Medications** | **Allergies** | **Family History** |
| Hypertension uncontrolled  Active smoker | No history of antiplatelet and antihypertension consumption | No history of allergies | No history of cardiac disease in his family |
| **Physical Examination** | | | |
| **General** | Patient is middle aged male, anxious | | |
| **HEENT** | Normocephalic and atraumatic, pupils equal round and reactive to light, extra ocular movements are fully intact, tympanic membranes clear, dry mucous membranes | | |
| **Neck** | Supple, no distended neck veins | | |
| **Lungs** | Vesicular | | |
| **Cardiovascular** | Sinus tachycardia, regular, no murmur or rubs | | |
| **Abdomen** | Soft, non-tender, and non-distended. No organomegaly. Normo-active bowel sounds | | |
| **Neurological** | Awake, following basic commands. Generally weak but symmetric strength in extremities. | | |
| **Skin** | Warm, wet. diaphoresis. No rash. | | |
| **GU** | Deferred | | |
| **Psychiatric** | Normal affect | | |

| Instructor Notes - Changes and CASE Branch Points | | |
| --- | --- | --- |
| **Intervention / Time point** | **Change in Case** | **Additional Information** |
| Within 10 minutes do not consult to cardiologist | Patient asking what happen to him and what is next plan form him | RN alerts the provider: “doctor, should we consult to cardiologist” |
| Consultation to cardiologist | Decision to primary PCI is made and followed by inform consent and inter-unit communication done by the ED head nurse |  |

**Ideal Scenario Flow**

Scene 1. Triage counter

At presentation, the patient was alert, warm extremities and rapid heart rate 100x/minutes. Triage officer identified the symptoms and subsequently, put the patient in the red zone, activate emergency medical officer and internal medicine resident and also the emergency nurse.

Scene 2.

The emergency team should do an initial assessment and initial treatment.

Patient’s blood pressure was 120/90 mmHg and oxygen saturation 99 % (O2 cannula 3 Liter/minutes)

ECG results: anterior septal STEMI

Consultation to interventional cardiologist for primary PCI done by the internist

Administrative preparation was performed by emergency nurse and head nurse

Transport preparation was done by emergency physician and emergency nurse

Laboratory results : full blood count, blood glucose, ureum and creatinine within normal limit

Scene 3.

Transport patient from ED to cath lab accompanied by 1 doctor, 1 nurse, and 1 transporter

Hand over the process to cath lab officer.

The patient put on catheterization laboratory table

No potential Branch Points

**Anticipated Management Mistakes**

- Not initiating inform concent : patient’s wife still refuse intervention, and agree primary PCI after proper information is given by internal medicine resident
- Not perform registration in ED : medical record will not available and doctors can not asking for laboratory examination
- Not perform registration in cardiac center : ED head nurse should contacted admission officer in cardiac center via mobile phone and giving information including patient name, medical record, and treatment plan (primary PCI)
