# Supplementary material for: ST-Elevation Myocardial Infarction: A Simulation Case for Evaluation of Interprofessional Performance in a Hospital
Source: Emerg Med Int. 2019 Oct 7;2019:7562637. doi: 10.1155/2019/7562637 (PMC6800974; doi:10.1155/2019/7562637)
Supplement: Supplementary Materials — S1: simulation case template; S2: visual stimulation (STEMI ECG); S3: evaluation sheet; S4: simulation video; S5: debriefing material. [file 7562637.f1.zip › 7562637.f1/S2 Visual stimulation (STEMI ECG).docx]

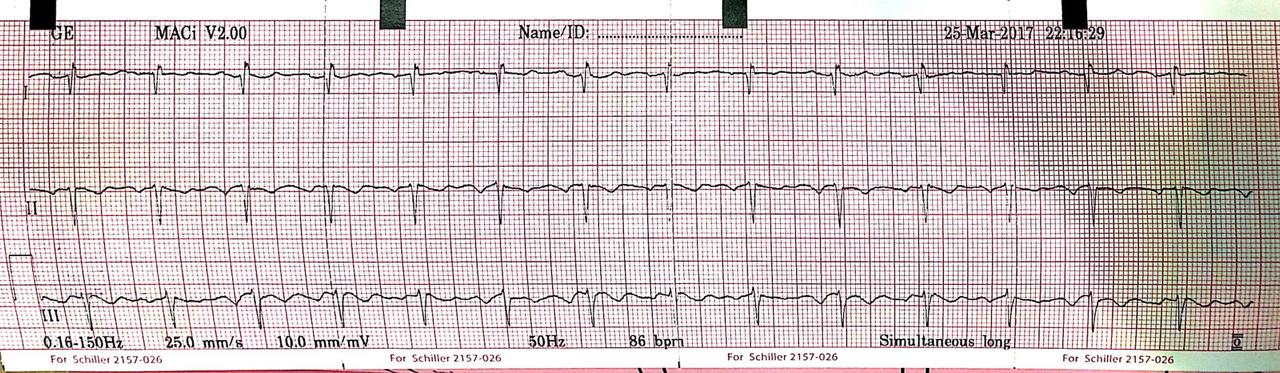
Appendix B. ECG for STEMI simulation (anteroseptal STEMI)


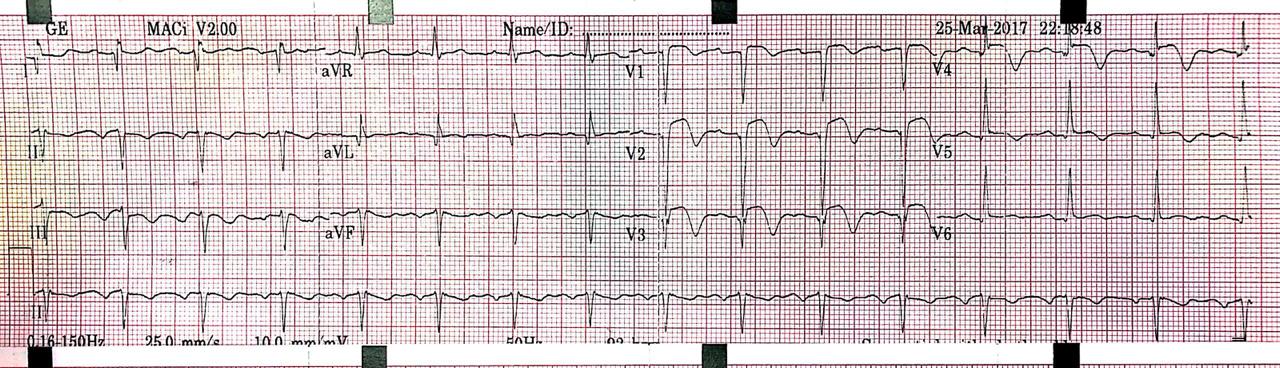


This is an author own image

(Hadiki Habib, Emergency Department Cipto Mangunkusumo Hospital, Jakarta-Indonesia.
