# Supplementary material for: ST-Elevation Myocardial Infarction: A Simulation Case for Evaluation of Interprofessional Performance in a Hospital
Source: Emerg Med Int. 2019 Oct 7;2019:7562637. doi: 10.1155/2019/7562637 (PMC6800974; doi:10.1155/2019/7562637)
Supplement: Supplementary Materials — S1: simulation case template; S2: visual stimulation (STEMI ECG); S3: evaluation sheet; S4: simulation video; S5: debriefing material. [file 7562637.f1.zip › 7562637.f1/S3 Evaluation sheet.docx]

Appendix C : Critical action check list

| Administration | Nurse | Emergency medical officer | time | Internal medicine resident |  |
| --- | --- | --- | --- | --- | --- |
|  | Triage  **Initial assessment in triage counter**   - Disposition to red zone |  |  |  |  |
| - registration was done by patient’s family | - **Completion of triage form in the red zone by triage nurse** - *Semifowler* position - Loosen patient’s clothes - Educate family to do registration process - Perform IV line cannulation (simulate the process) - Taking blood sample from the vein (simulate the process) | - **Receiving patient in the red zone** - Activate internal medicine resident to join because patient has typical chest pain - Perform ECG testing - Perform oxygen therapy (nasal cannula) - *reassurance* - placing cardiac monitor - Nitrate 5 mg sublingual extra - Aspirin 320 mg tablet - Clopidogrel 300 mg tablet - Morphin 2-4 mg intra-vein bolus |  | - Evaluation of chief complain (typical and progressive chest pain - Declaration that patient has acute coronary syndrome |  |
| - Medical record and identity sticker is available in the red zone - customer service officer performed education of hospital regulation for patient’s wife - admission and insurance activation process finish | - put on identity bracelet - write on nurse assessment in medical record - sending blood sample to the emergency laboratory | - write on medical assessment, diagnosis and plan of action in the medical record |  | - **ECG interpretation** - Write online receipt for patient medication - Consultation to cardiologist on call to report the STEMI diagnosis and request for the primary PCI - Agreement from the cardiologist consultant about patient diagnosis and decision to activate cardiac laboratory - Write on inpatient instruction so that the patient can be send to cardiovascular care unit after primary PCI - Write on consultation form to cardiac laboratory |  |
| - Admission officer in cardiac center call the cardiac laboratory officer and interventional cardiologist to confirm that they ready to perform primary PCI - ED head nurse receive call from cardiac laboratory that they ready to perform primary PCI and the patient can be send immediately | - Emergency nurse report to the head nurse that the patient is planning to undergo primary PCI - ED head nurse call admission officer and and cardiac laboratory officer to confirm that the patient is ready to send   **Critical ill transport preparation**  Transport form is completely fill  Oxygen transport device  Transporter officer  Cardiac monitor | - Medical doctor perform inform consent to the patient and wife about indication, benefit and risk of primary PCI - **Patient and family agree to undergo primary PCI, and completely fill inform consent form.**     **Advance medical treatment in ED prior to primary PCI** :   - Bed side focused echocardiography - Clopidogrel 300 mg prior primary PCI - Heparin 60 unit/kgbb IV , followed by continuous heparin 12 unit/Kg/hours or enoxaparin 30 mg iv - B blocker for anti-ischemia of no pulmonary oedema documented   Bisoprolol 2.5-5 mg oral   - ACE inhibitor - Statin   Atorvastatin 40 mg oral or  Rosuvastatin 20 mg oral | | | |
| - Patient’s family come to the admission room of cardiac center to fill in administration form | - **Patient arrive at main entrance of cardiac laboratory** - **Handover between ED officers (doctor and nurse) to cardiac laboratory nurse** - **Patient enter the cardiac laboratory room** - **Simulation end** | |  | - Interventional cardiologist is present in cardiac laboratory |  |
