# Supplementary material for: ST-Elevation Myocardial Infarction: A Simulation Case for Evaluation of Interprofessional Performance in a Hospital
Source: Emerg Med Int. 2019 Oct 7;2019:7562637. doi: 10.1155/2019/7562637 (PMC6800974; doi:10.1155/2019/7562637)
Supplement: Supplementary Materials — S1: simulation case template; S2: visual stimulation (STEMI ECG); S3: evaluation sheet; S4: simulation video; S5: debriefing material. [file 7562637.f1.zip › 7562637.f1/S5 Debriefing material.docx]

Appendix D. Debriefing Material and Recommendation

| Finding | Recommendation |
| --- | --- |
| Door-to-ECG time 8 minutes  Door-to-Skin puncture time 110 minutes  Delay triage response for the next patient  Long wait in patient admission  Long wait for oral medication  Several attempts for  telephone consultation to cardiologist  prolonged admission process in cardiac laboratory  No confirmation for inpatient process in coronary care unit after intervention | Target accomplished  During simulation, only one ECG available for the red and yellow zone, this condition contributes to diagnosis delay. We propose to use three ECG in every zone in emergency department (red, yellow and green).  Target not accomplished  We propose to use a standardized tool called ‘5 D times’ to evaluate performance of the system in managing STEMI  We propose 2 persons as triage officer every shift, due to the importance of triager to perform rapid assessment and response, and to handle patient arrival in our ED almost every 10 minutes  Admission was done by patient’s family and it took almost 10 minutes in every 1 admission process, it is very important to put the patient data in electronic health record because to obtain pharmacological treatment and devices, we need to make an online order.  Rapid pharmacological treatment such as sublingual Nitrates, Aspirin, Clopidogrel, and Morphine are the cornerstone of acute cardiac care, during simulation, it took 20 minutes to get the medication on board, we propose an emergency list of device and medication for the emergency order before the electronic health record could be accessed.  The consultation was done by telephone, the problem was sometimes the consultant did not receive the call, so the registrar should divert the call to another consultant.  Family of the patient was asked to come to cardiac building and bring all the paper of insurance and the consultation letter from ED, the process had consumed most of the time in managing patient in ED, we propose to use telemedicine such as WhatsApp group to communicate between unit without asking the family to walk around the hospital for administration tasks.  There was no agreement reached for the patient next care after the intervention, mostly, the patient bounced back to ED, this will lead to ED condensation and reduce the patient safety for the post-intervention cases. |
